# A Precision Medicine Approach to Biomarker Utilization in Pediatric Sepsis-Associated Acute Kidney Injury

**DOI:** 10.3389/fped.2021.632248

**Published:** 2021-04-14

**Authors:** James D. Odum, Hector R. Wong, Natalja L. Stanski

**Affiliations:** ^1^Division of Critical Care, Cincinnati Children's Hospital Medical Center, Cincinnati, OH, United States; ^2^Department of Pediatrics, University of Cincinnati College of Medicine, Cincinnati, OH, United States

**Keywords:** sepsis, acute kidney injury, biomarkers, precision medicine, enrichment

## Abstract

Sepsis is a leading cause of morbidity and mortality in critically ill children, and acute kidney injury (AKI) is a frequent complication that confers an increased risk for poor outcomes. Despite the documented consequences of sepsis-associated AKI (SA-AKI), no effective disease-modifying therapies have been identified to date. As such, the only treatment options for these patients remain prevention and supportive care, both of which rely on the ability to promptly and accurately identify at risk and affected individuals. To achieve these goals, a variety of biomarkers have been investigated to help augment our currently limited predictive and diagnostic strategies for SA-AKI, however, these have had variable success in pediatric sepsis. In this mini-review, we will briefly outline the current use of biomarkers for SA-AKI, and propose a new framework for biomarker discovery and utilization that considers the individual patient's sepsis inflammatory response. Now recognized to be a key driver in the complex pathophysiology of SA-AKI, understanding the dysregulated host immune response to sepsis is a growing area of research that can and should be leveraged to improve the prediction and diagnosis of SA-AKI, while also potentially identifying novel therapeutic targets. Reframing SA-AKI in this manner – as a direct consequence of the individual patient's sepsis inflammatory response – will facilitate a precision medicine approach to its management, something that is required to move the care of this consequential disorder forward.

## Introduction

Sepsis is common in the pediatric intensive care unit (PICU), accounting for 75,000 hospitalizations annually in the United States (1). Children with sepsis suffer substantial morbidity and mortality, and those risks are further increased by the co-incidence of acute kidney injury (AKI), a frequent complication of pediatric sepsis (2, 3). Impacting almost half of critically ill children who meet criteria for severe sepsis (4), sepsis-associated AKI (SA-AKI) has been associated with poor outcomes including prolonged lengths of stay, higher mortality, and increased healthcare costs (3, 5, 6). Unfortunately, despite the burden it imposes on health outcomes, there are currently no effective disease-modifying therapies for SA-AKI once present (7, 8). As a result, therapeutic approaches are centered on prevention and supportive care, including employment of renal protective measures and timely initiation of renal replacement therapy, if indicated (9, 10).

A primary limitation to the successful development of novel therapies to treat SA-AKI is an incomplete understanding of the pathophysiology. Historical perspectives considered SA-AKI to be the result of renal hypoperfusion during the shock state, leading to acute tubular necrosis (11). However, the modern pathophysiologic model of SA-AKI recognizes a much more complex and heterogeneous disease – a combination of a dysregulated host inflammatory response to infection, altered microcirculatory blood flow, and metabolic derangements that ultimately lead to cell cycle arrest and apoptosis of renal tubular epithelial cells (12, 13). Difficulties in treatment development encountered due to this complexity are further exacerbated by the limited diagnostic strategies for SA-AKI, as it is now well-established that serum creatinine and urine output – the current gold standards for diagnosis – are fraught with issues, particularly among patients with sepsis (14). Indeed, the limitations of these tools are highlighted by the recent 23rd Acute Dialysis Quality Initiative recommendations, which suggest incorporating tubular injury biomarker status into the definition of AKI, when available (15). Taken together, the lack of precision diagnostics for SA-AKI, coupled with a limited understanding of the individual heterogeneous pathophysiology, have prevented advances in therapy beyond our current standards of prevention and supportive care (16).

With these issues in mind, it is not surprising that there is a growing interest in identifying biomarkers for SA-AKI. In addition to the clear need for validated injury biomarkers to improve diagnostic precision once present, biomarkers that allow for early identification of patients at risk for severe, persistent SA-AKI and those that reflect the patient-specific underlying pathophysiology are needed, as they might allow for prompt implementation of renal protective strategies, identification of biologically important targets for development of novel therapies, and provide a mechanism for enrichment of future clinical trials (17). Given the complexity of SA-AKI outlined above, it is unlikely that one biomarker measured at one moment in time will be able to achieve these goals, and this reality should inform our approach to identifying and employing biomarkers for SA-AKI.

This mini-review proposes a new framework for the discovery and utilization of biomarkers for SA-AKI. The foundational premise is that the pathophysiology of SA-AKI is directly tied to an individual's unique sepsis-related inflammatory response, and thus the diagnostic and treatment approach to SA-AKI may be different from other forms of AKI. Within this framework, we will briefly describe the current state of biomarkers for SA-AKI and discuss their limitations. We will then evaluate how biomarkers have been employed for the identification of individual sepsis molecular signatures, and how these may be leveraged in SA-AKI. Ultimately, if biomarkers can be biologically linked to the dysregulated inflammatory response to sepsis, then a precision medicine approach to the diagnosis and treatment of SA-AKI can be utilized to improve patient outcomes.

## The Current Application of Biomarkers in Sepsis-Associated AKI

To date, a variety of biomarkers for SA-AKI have been studied. The biological underpinnings of these biomarkers vary, and include direct markers of tubular injury, regulatory proteins responsible for promoting cell cycle arrest, and more recently, proteins involved in the inflammatory cascade induced by sepsis (18, 19). Thus, far, SA-AKI research utilizing biomarkers has been limited to improving diagnostic and predictive capacity, most with modest success (18). Importantly, there have been no interventional trials to date utilizing biomarkers to initiate disease-specific therapy in SA-AKI. An overview of the biomarkers that have been most widely investigated in SA-AKI is included in Table 1.While the purpose of this review is not to cover these previously studied biomarkers of SA-AKI in detail, two deserve more in depth discussion.

**Table 1:** Investigated biomarkers of sepsis-associated acute kidney injury.

**Biomarker**	**Site of production**	**Function**	**Pathophysiology**	**Measured**	**Potential applications in SA-AKI**	**Time to AKI**	**Limitations**
Neutrophil gelatinase-associated lipocalin (NGAL)	Systemic: liver, circulating neutrophils, epithelial cells	Binds bacterial siderophores to inhibit growth; also has anti-apoptosis effects and enhances proliferation of renal tubules (20)	Marker of renal tubular epithelial injury and systemic inflammation (20)	Plasma, Urine	Plasma NGAL within 24 h of admission predicted SA-AKI in children with an AUROC of 0.68 (21)	AKI diagnosed by day 7 (median 1, range 1–6) (21)	High sensitivity with poor specificity
	Kidney: proximal tubule, thick ascending limb of Henle's loop, distal tubule, and collecting duct				Meta-Analysis: plasma NGAL predicted SA-AKI with an AUROC of 0.86, and urine NGAL with an AUROC of 0.90 (22)		Elevated in the setting of systemic inflammation
Kidney injury molecule-1 (KIM-1)	Kidney: tubular apical transmembrane protein, soluble form excreted in urine	Involved with repair of renal tubular epithelial cells (23)	Upregulated during ischemic and nephrotoxic AKI (23)	Urine	Increased within 6–24 h of admission in patients with SA-AKI. Level at 24 h predicted SA-AKI with an AUROC of 0.91 (24)	AKI diagnosed by 48 h (24)	Limited investigations in pediatric SA-AKI
Netrin-1	Systemic: nervous system, heart, lung, liver, intestines, blood vessels	Axon guidance molecule, inhibits leukocyte migration, promotes endothelial chemoattraction (18)	Increased production in renal tubular epithelial cells in response to ischemic AKI (18)	Urine	Levels peaked early, within 3-6 h of admission, in patients SA-AKI. Level at 3 h predicted SA-AKI with an AUROC of 0.86 (24)	AKI diagnosed by 48 h (24)	Limited investigations in pediatric SA-AKI
	Kidney: secreted by proximal tubule epithelial cells, present in renal microvascular endothelial cells						
Tissue inhibitor of metalloproteinase-2 (TIMP-2)	Renal tubular epithelial cells	Promotes G1 cell cycle arrest via increasing p27 expression (25)	In response to tubular epithelial damage, TIMP-2 and IGFBP7 expression is increased to initiate cell cycle arrest and signal to neighboring cells via paracrine and autocrine modalities (26)	Urine	Product of urine TIMP-2 · IGFBP7 predicts SA-AKI within 12 h of admission with an AUROC of 0.84 (27)	AKI diagnosed within 12 h of study enrollment (28)	Limited study in children, FDA approval does not apply to patients <18 years old
Insulin-like growth factor-binding protein 7 (IGFBP7)	Renal tubular epithelial cells	Promotes G1 cell cycle arrest via increasing expression of p53 and p21 (25)			Now available as FDA approved tool known as NephroCheck^®^ in adults with one or more AKI risk factors, including sepsis (27)		
Soluble triggering receptor expressed on myeloid cells 1 (sTREM-1)	Systemic: expressed by neutrophils and monocytes	TREM-1 triggers secretion of pro-inflammatory mediators in response to extracellular bacterial infections (29). sTREM-1 is a soluble form of TREM-1 that modulates cytokine production to prevent hyper-responsive inflammatory cascade (30)	Plasma sTREM-1 levels strongly correlate to sepsis severity (31). It may be filtered into the urine, or produced and excreted locally during acute tubular necrosis (32)	Plasma, Urine	Plasma sTREM-1 predicted SA-AKI with an AUROC of 0.746 and urine sTREM-1 with an AUROC of 0.778 24-h prior to diagnosis by SCr (33)	AKI diagnosed by day 7 (median 2, range 1–7) (33)	No prospective studies in pediatric SA-AKI
	Kidney: endothelial cells, tubular epithelial cells, infiltrating inflammatory cells				Urine sTREM-1 increased 48-h prior to SA-AKI in adults (34)		
Interleukin-18 (IL-18)	Systemic: secreted by macrophages after precursor is cleaved by caspase-1 intracellularly	IL-18 is a proinflammatory cytokine that induces interferon gamma production from natural killer cells, also induces T-cells to produce interleukin-17 (35)	IL-18 is released by renal tubular cells in response to injury and is thought to mediate acute tubular necrosis (36)	Urine	Urinary 1L-18 increased 24–48 h prior to diagnosis of AKI in adult patients with Acute Respiratory Distress Syndrome and AKI. IL-18 demonstrated an AUC of 0.73 to predict AKI in the next 24 h (36)	AKI diagnosed by day 6 of hospitalization. Biomarker values reported 24 h prior to time AKI was diagnosed	No studies in children, less specific to acute kidney injury
	Kidney: released in response to tubular injury						

*AKI, acute kidney injury; SA-AKI, sepsis-associated acute kidney injury; AUROC, area under the receiver operating curve; FDA, Food and Drug Administration; TREM-1, triggering receptor expressed on myeloid cells 1; SCr, serum creatinine*.

### Neutrophil Gelatinase-Associated Lipocalin (NGAL)

As the most widely studied biomarker of AKI, NGAL – a protein produced by the injured nephron that can be measured in both urine and serum – has also been studied extensively as a biomarker of SA-AKI (37). While NGAL has been shown to successfully identify patients with AKI secondary to a variety of etiologies (38–40), its utility in sepsis is less clear (41–44). This is in large part due to an increase in systemic NGAL production – namely by neutrophils and the liver – as part of the inflammatory response to infection, independent of injury to nephrons (20). The consequence of this lack of kidney-specific production of NGAL has been modest performance when utilized for diagnosis and prediction of SA-AKI, often with high sensitivity but poor specificity (Table 1) (21, 22). Unfortunately, difficulty disentangling the fraction of NGAL elevation that is attributable to AKI, vs. a more generic systemic inflammatory response among patients with sepsis, likely limits its utility as a single biomarker for the diagnosis of SA-AKI, although more study is warranted.

### Cell Cycle Arrest Markers

The induction of cell cycle arrest in renal tubular epithelial cells plays an important role in the early pathophysiology of all forms of AKI (11). Consequently, the expression of cell cycle arrest proteins tissue inhibitor of metalloproteinase-2 (TIMP-2) and insulin-like growth factor-binding protein 7 (IGFBP7) have been shown to be increased in renal tubular cells in response to stress or injury (18, 25). The combination of TIMP-2 and IGFBP7 for the prediction of AKI in high risk patients has been examined in several landmark studies (Table 1) (26, 28, 45), and is now approved by the U.S. Food and Drug Administration for critically ill adults with one or more risk factors for AKI, including sepsis (25). In adults with sepsis, this tool (known as NephroCheck^®^) demonstrated an area under the receiver operating curve (AUROC) of 0.84 for the prediction of SA-AKI, and its predictive performance significantly improved via the addition of a clinical prediction model (AUROC of 0.94) (27). The use of NephroCheck^®^ to assess the impact of directed implementation of standardized renal protection strategies compared to standard of care in patients with septic shock will be assessed in the upcoming Limiting AKI Progression in Sepsis (LAPIS) Trial (NCT04434209) (46). Unfortunately, this tool has not been studied robustly nor been validated in children.

As noted above and in Table 1, there are several limitations to the use of these biomarkers in pediatric SA-AKI. First and foremost, the data for their use in pediatric sepsis is scare, and this is especially problematic given a growing host of literature to suggest fundamental differences in the sepsis inflammatory response – and thus, the risk of SA-AKI – based on age (47–50). Furthermore, the biologic action of many of these biomarkers appear to be non-specific to sepsis (38, 51, 52), thereby providing no information regarding the patient's underlying inflammatory state, which is likely necessary to identify effective, patient-specific therapies for SA-AKI. Taken together, these realities suggest that additional approaches to biomarker discovery and utilization is required.

## The Sepsis Molecular Signature and Its Role in Sepsis-Associated AKI

As outlined above, sepsis is a complex syndrome that stems from a dysregulated host immune response to an infectious trigger, and is a leading cause of death and disability in critically ill children (53). Given these consequences, substantial resources have been focused on improving the care of patients with sepsis, however, these efforts have failed to produce meaningful therapeutic advances beyond the mainstays of supportive care and antibiotics (54). Failures are undoubtedly tied to the heterogeneity of the disease expression on the individual patient level (17). As such, attempts to resolve this heterogeneity by identifying the sepsis molecular signature of a patient are becoming more common, as successful strategies for doing so could allow for more targeted employment of therapies (55–61).

**Figure 1:**
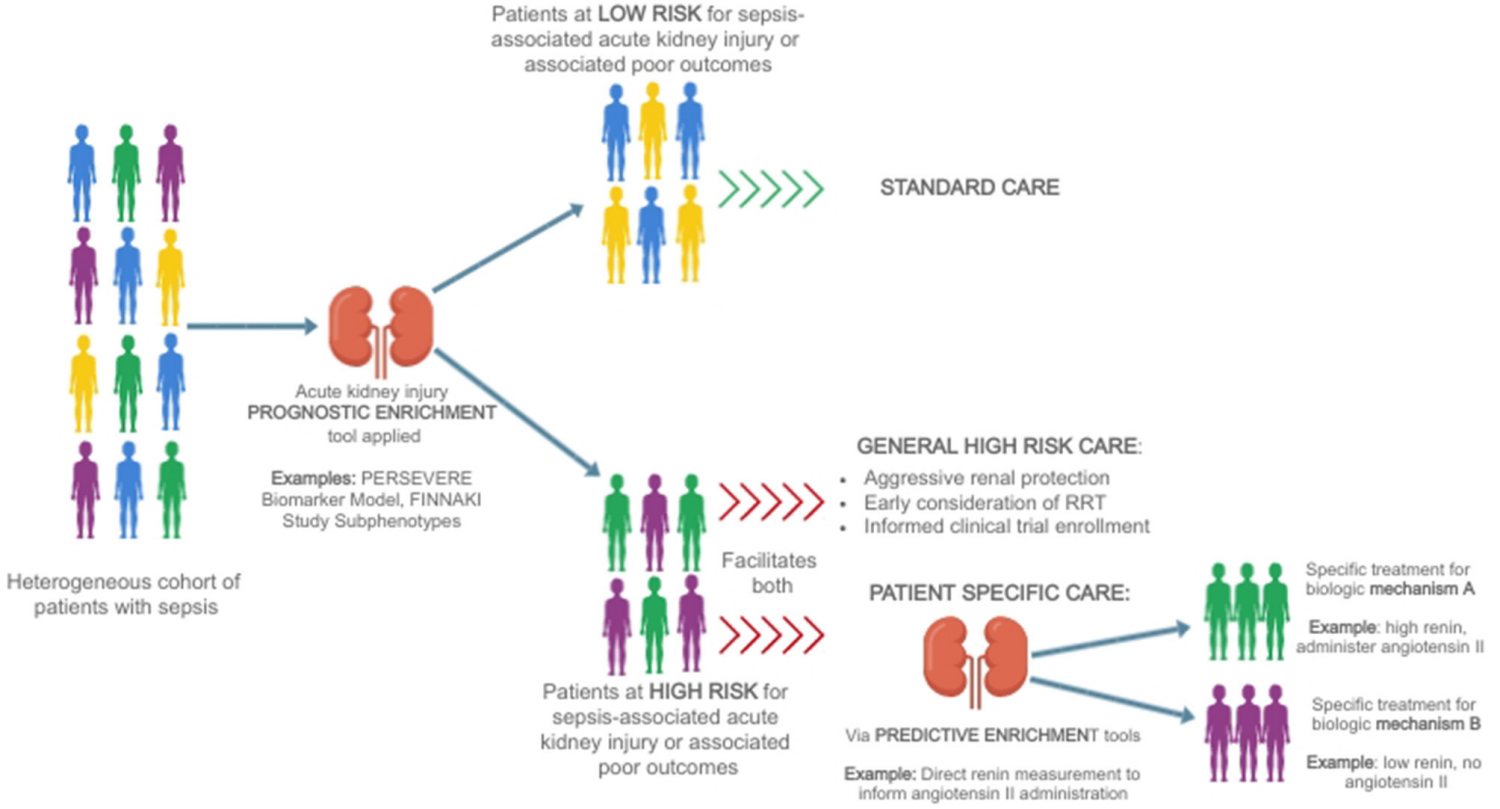
A heterogeneous group of patients with sepsis is divided first using a prognostic enrichment tool into those at high and those at low risk of sepsis-associated acute kidney injury [for example, the PERSEVERE Biomarker Model (64)] or associated poor outcomes such as delayed renal recovery or mortality [for example, FINNAKI Study subphenotypes (65)]. Patients who are at low risk may be treated with standard therapy, while patients at high-risk may receive more aggressive care aimed at renal protection, earlier consideration of renal support in the form of renal replacement therapy, and be considered for informed enrollment in clinical trials aimed at identifying therapies for sepsis-associated acute kidney injury. Ultimately, the goal is to utilize predictive enrichment tools to further subdivide patients on the basis of biology [for example, using direct renin levels to inform the use of angiotensin II (66)], allowing for the implementation of patient-specific therapies.

This concept of separating a heterogeneous group of patients into more homogenous subgroups to guide management is termed *enrichment*, a fundamental tenant of precision medicine (62). *Prognostic enrichment* refers to selecting a subgroup of patients who share a similar likelihood of suffering an outcome of interest, such as mortality, while *predictive enrichment* selects a subgroup who are more likely to respond to a particular therapy based on underlying biology (63). This general concept, and how it may be employed to direct a precision medicine approach to SA-AKI therapy, is depicted in Figure 1.

In pediatric sepsis, *prognostic enrichment* strategies have been used to develop a set of serum biomarkers – known as the Pediatric Sepsis Biomarker Risk Model (PERSEVERE) – capable of reliably assigning baseline risk of 28-day mortality (67). This model incorporates five serum protein biomarkers measured in the first 24 h of septic shock that were originally identified utilizing discovery-oriented genome-wide profiling of children with septic shock (68, 69), and then narrowed further using classification and regression tree (CART) modeling for estimation of baseline mortality risk (67). An updated version of the model (PERSEVERE-II) incorporates platelet count, and has been recently prospectively validated for the prediction of mortality (70). Similarly, *predictive enrichment* has also been utilized to subgroup patients based on gene expression, which led to the identification of two distinct endotypes that may require different treatment approaches (58). For example, one particular endotype – endotype A – is associated with increased repression of genes that regulate adaptive immunity and glucocorticoid receptor signaling, and patients with this endotype have demonstrated an increased mortality rate when treated with corticosteroids (71, 72). This association between endotype A and poor outcome in response to corticosteroids was recently corroborated among adults with septic shock (73). Given our current understanding of the significant role that the host inflammatory response plays in the pathophysiology of SA-AKI (12, 13), it is reasonable to consider leveraging this pediatric sepsis enrichment work to improve the care of SA-AKI, a similarly heterogeneous disorder (11, 12).

## The Goal: Sepsis-Specific Biomarkers for a Precision Medicine Approach to Sepsis-Associated AKI

A precision medicine approach to SA-AKI will require both prognostic enrichment tools to identify high risk patients early and accurately, and predictive enrichment tools to deliver the right treatment to the right patient. Biomarkers play an important role in achieving these goals, however, we believe that a shift to include biomarkers of the dysregulated immune response to infection is prudent. Such a shift will also require a reframing of AKI in sepsis, recognizing that it is not simply “associated” with sepsis (as suggested by the term SA-AKI), but a disease state that is induced by the host inflammatory response. In this section, we will outline the current application of precision medicine to the study of SA-AKI within this framework, and highlight the remaining critical knowledge gaps.

### Prognostic Enrichment Tools for SA-AKI

The first step to improving outcomes for patients with SA-AKI is early identification of those at highest risk. While sepsis is perhaps the most significant risk factor for AKI in critically ill patients, a significant proportion of patients with septic shock do not develop AKI. Therefore, further delineation of an individual's risk profile via the development of prognostic enrichment tools is required. To date, few validated prognostic enrichment strategies for SA-AKI that incorporate sepsis-specific biomarkers exist, as outlined below.

Leveraging work done in the more advanced field of sepsis precision medicine, researchers have utilized “omic” technologies (notably genomics, transcriptomics and proteomics) to identify patients at high risk for persistent SA-AKI (10, 64, 74). Using microarray technology to study SA-AKI related transcriptomics, one group retrospectively identified 21 candidate biomarkers for the prediction of SA-AKI based on the upregulation of mRNA gene probes in patients with persistence of severe SA-AKI at day 7 of septic shock (74). The expression pattern of these 21 upregulated genes were shown to predict the presence of this severe, persistent form of SA-AKI with high sensitivity (98%) and reasonable specificity (80%) (74). Results from this work informed a second study in which the protein products of five of the aforementioned 21 genes–elastase 2 (ELA2), fibroblast growth factor 13, matrix metalloproteinase 8 (MMP8), olfactomedin 4 (OFM4), and proteinase 3 (PRTN3) – were incorporated into a new CART-derived model to predict the presence of SA-AKI at day 3 of septic shock (10). The test characteristics of this model in the derivation cohort were robust, with an AUROC of 0.95; when tested in a validation cohort, the predictive capacity of the model remained reasonable with an AUROC of 0.82, which was superior to knowledge of AKI stage by serum creatinine on the day of septic shock development alone (AUROC 0.73) (10). Using a similar approach, a more recent study utilized the PERSEVERE biomarkers and AKI stage by serum creatinine on the day of admission to develop a model for prediction of severe SA-AKI at day 3. This model had similarly impressive test characteristics, with an AUROC of 0.95 (64). Unfortunately, while these models represent potentially feasible prognostic enrichment tools for SA-AKI, they have not yet been prospectively validated nor utilized to inform patient care, which represent areas of future study.

Another strategy incorporating biomarkers that has been utilized for prognostic enrichment in SA-AKI is latent class analysis (LCA). This approach allows for the incorporation of multiple variables– including comorbidities, clinical data and biomarkers – to allow for the identification of potential subphenotypes of heterogeneous disease states. Using this methodology, a recent *post-hoc* analysis of the FINNAKI Study described two subphenotypes of critically ill patients with SA-AKI who have significantly different rates of mortality and renal recovery (65). Patients categorized as subphenotype 2 – which was associated with increased mortality and decreased short-term renal recovery – demonstrated elevations in biomarkers associated with endothelial dysfunction and an overall increased inflammatory state. Interestingly, four of the significantly upregulated inflammatory biomarkers in subphenotype 2 (ELA2, OFM4, MMP8 and PRTN3) overlapped with the above mentioned AKI prediction model derived by Wong and colleagues (10). Using a similar approach, a second group also identified two SA-AKI subphenotypes (AKI-SP1 and AKI-SP2) via the application of LCA to a panel of 29 clinical and biomarker variables (75). This study similarly showed decreased survival and renal recovery in patients with upregulation of biomarkers associated with endothelial dysfunction and inflammation, although the included biomarkers differed. While these LCA-driven studies identified high-risk subphenotypes of patients already known to have SA-AKI, they represent potentially viable prognostic enrichment tools, specifically to help delineate patients most likely to benefit from enrollment in clinical trials, as well as from potentially high-risk and high-resource utilizing therapies such as renal replacement therapy (RRT).

### Predictive Enrichment Tools for SA-AKI

The identification of predictive enrichment tools for SA-AKI–those that provide insight into the underlying pathophysiology and thereby reveal potential treatment strategies–remains an elusive goal. Predictive enrichment tools are particularly helpful in heterogeneous disease states, as they may identify subphenotypes of patients who might benefit from a specific, biologically-based therapy. While Figure 1 outlines an ideal circumstance in which predictive enrichment occurs in an identified high risk subset of patients, it is important to note that the development and use of predictive enrichment tools does not necessarily rely on the availability of reliable prognostic enrichment strategies. However, the identification of effective predictive enrichment tools requires a deep understanding of the patient-specific pathophysiology, which remains a significant barrier in SA-AKI.

Thus far, the only proposed predictive enrichment strategy that is clinically feasible was elucidated via a series of *post-hoc* analyses of the Angiotensin II for the Treatment of High-Output Shock (ATHOS-3), a clinical trial of adults with vasodilatory shock treated with angiotensin II (76). In these studies, the authors were able to demonstrate that patients who were treated with angiotensin II had improved 28-day survival and earlier discontinuation of RRT (77), and that these advantages were best seen in patients who had higher serum renin levels prior to angiotensin II administration, suggestive of sepsis-induced angiotensin converting enzyme deficiency (and thus angiotensin II deficiency) in the setting of endothelial injury (66). From these findings, the authors postulated that administration of exogenous angiotensin II to patients with vasodilatory shock may be beneficial beyond simply increasing blood pressure, as it was also demonstrated to normalize high renin levels, which have been known to be proinflammatory (66). Given that serum renin levels can be easily measured, this example of predictive enrichment can and should be applied prospectively in future studies examining the effect of angiotensin II on mitigation of SA-AKI.

## Conclusion

SA-AKI is a common and consequential diagnosis in critically ill children, yet successful diagnostic and treatment strategies remain unacceptably scarce. In order to improve the care of patients with SA-AKI, researchers must move toward a precision medicine approach that considers the heterogeneity of the disease on the individual patient level. While biomarkers will undoubtedly play an important role in these endeavors, the complex pathophysiology of SA-AKI requires that we consider the use of biomarkers specific to the individual sepsis inflammatory response, a key driver of renal injury in these patients. To do this, researchers must leverage and build upon existing sepsis precision medicine work, facilitating the development of prognostic and predictive enrichment tools that could advance the care of SA-AKI beyond prevention and renal support. A necessary and feasible first step in this process is the development and validation of reliable tools for the prediction of patients at highest risk for SA-AKI, as such a tool could facilitate the implementation of early and aggressive renal protection strategies, and perhaps more importantly in pediatrics, reduce the number of patients needed to study by informing enrollment in clinical trials aimed at identifying disease-modifying therapies. While the use of individual patient biology-driven therapies via predictive enrichment remains an elusive goal, reframing SA-AKI as a heterogeneous disease that will likely require an individualized approach to therapy is an important first step that should inform future research.

## Author Contributions

JO, NS, and HW were responsible for writing and editing this mini-review. All authors approved the final version for submission.

## Conflict of Interest

The Cincinnati Children's Research Foundation has submitted a provisional patent application for the sepsis-associated acute kidney injury prediction strategy discussed in this review article. NS and HW were named as co-inventors in the patent application. The remaining author declares that the research was conducted in the absence of any commercial or financial relationships that could be construed as a potential conflict of interest.
